# Approach for the structural reliability analysis by the modified sensitivity model based on response surface function - Kriging model

**DOI:** 10.1016/j.heliyon.2022.e10046

**Published:** 2022-08-05

**Authors:** Lin Zhu, Jianchun Qiu, Min Chen, Minping Jia

**Affiliations:** a School of Mechanical Engineering, Yangzhou University, Yangzhou 225001, China; b School of Advanced Technology, Xi'an Jiaotong-Liverpool University, Suzhou 215123, China; c School of Mechanical Engineering, Southeast University, Nanjing 211189, China

**Keywords:** Reliability, Kriging model, Sensitivity, Working conditions, Multiple coupling parameters

## Abstract

The sensitivity analysis model is widely used to describe the impacts of condition parameters on structural reliability. However, the classical sensitivity analysis model is limited to the small number of influence parameters and has no high prediction accuracy. Integrating the response surface function - Kriging model with Sobol sensitivity algorithm, a revised sensitivity model is proposed in this paper. And the quantitative sensitivity analysis for the influence of condition parameters on structural reliability are achieved through combining the revised sensitivity model with the experimental design of coupling parameters, range verification, the multi-body dynamics analysis and the structural statics analysis. The proposed analysis model is mainly applied in large structures with multiple influence parameters. Finally, a typical port crane is adopted to verify the accuracy and effectiveness of the proposed model. The results reveal that among the multiple parameters, the biggest sensitivity influence is the trolley position, while the least one is the lifting speed. The average prediction accuracy of the quantitative structural reliability index for the influencing parameters is up to 95.91%. The revised sensitivity model enables the accurate assessment of structural relativity with plenty of coupling condition parameters.

## 0 Introduction

Large mechanical equipment is developing in the direction of intelligent operation, lightweight structure and large-scale operation, and its operation safety is becoming more and more prominent [1], [2], [3]. With the increase of service time, tiny cracks may bring huge risks to the structure safety. The complicate operating conditions and external random parameters become more and more significant for the structural reliability [4], [5], [6] and operation safety [7], [8]. Therefore, the quantitative identification of the reliability influence degree becomes the research focus [9], [10].

In industrial applications, the structure reliability is normally affected by lots of parameters [11], [12], [13], [14], [15], [16], [17], [18]. The coupled effects could be crucial for the safe operation. For optimized operation settings of a brake system, Du [19] considered the relation between the operation parameters and working conditions. Wooram [20] used finite element simulation to analyze the dynamic failure of both similar and different combinations of spot welded automotive steel plates. Taking the mechanical properties as the reference condition and considering the influence of other conditions, the dynamic lap shear and coach peeling sample tests are carried out to evaluate the dynamic failure behavior. Heredia [21] proposed a nonparametric prediction approach for the influence of key parameters on output parameters, based on the bootstrap-based bias correction theory and the aggregated first-order index. Ren [22] established a revised analysis model by the partial differential constraint equations to sort the operation conditions. Then a memetic algorithm was proposed to achieve the high-precision results. The validation case also illustrated the computational efficiency. However, the above research only considers the qualitative influence relationship of individual factors, and does not fully consider the quantitative influence degree of parameters.

Kriging optimization algorithm is widely applied in multidisciplinary optimization design problems for complex systems [23], [24], [25], [26], [27], [28], [29]. It can accurately converge to the real optimal solution, and has the ability of accurate constraint processing. Costas [30] enhanced the performance of frontal impact absorber through multi-objective optimization technology by nearly 50%, and validated result by the drop tower test. Zhang [31] proposed a new penalty blind likelihood Kriging method, which uses the grid search of cross validation to select good regularization parameters. The effectiveness was verified by two engineering examples. Mallik [32] proposed an aeroelastic gust response analysis method at high angle of attack based on Kriging method, and verified its accuracy through many examples. Deng [33] used the multi-objective optimization design method to study the crash worthiness of the system, and used the Kriging method to construct the alternative model of specific energy absorption (SEA) and initial peak force. However, Kriging surrogate model only considered the fitting of the influence degree of individual parameters, and does not deeply investigate the quantitative relationship of global sensitivity between different parameters.

Sensitivity analysis, an approach to quantify the influence levels under complex working condition, plays an important role in the in-depth study of reliability indicators [34], [35], [36], [37], [38], [39], [40]. Lu [41] proposed a reliability sensitivity model using the moment-based saddle point approximation theory and the random perturbation technology, through which the influence impact of random variables on the output parameter were quantified. Xiao [42] studied the local sensitivity analysis by proposing a direct integration method based on Sobol' theory. Through the direct integration of the optimal polynomial model, the accurate first-order and interactive sensitivity index can be obtained. Liu [43] proposed a new moment-independent sensitivity index to quantify the impact of individual input variable on the dependent output parameter of model. A multi-response Gaussian process (MRGP) proxy method with separable covariance estimated the multi-response and the sensitivity index effectively. Zhang [44] studied the sensitivity influence of the input variable distribution parameters on structural failure probability based on the Kriging model. The newly developed sensitivity index was compared with the classic sensitivity index to illustrate the effectiveness and accuracy.

Literature [41], [42], [43], [44] can quantitatively realize the sensitivity relationship between influencing parameters and structural reliability. However it lacks the consideration of global sensitivity, without considering the integrated effects of external influencing parameters [45], [46], [47], [48], [49], [50], [51]. It is challenging to evaluate the structural reliability under complex conditions precisely. Not only the local sensitivity, but also the global sensitivity should be taken into account.

Integrating the optimal response surface function - Kriging model and the Sobol sensitivity model, this paper proposed a revised sensitivity model to investigate the structural reliability comprehensively. And the quantitative sensitivity analysis results of various coupling influence parameters are achieved through the experimental design of coupling parameters, the range verification, the multi-body dynamics analysis, the structural statics analysis and the revised sensitivity calculation. Finally, the feasibility and accuracy of the proposed approach are verified by an industrial case.

## 1 Sensitivity model based on response surface function-Kriging model

### 1.1 Response surface function - Kriging model

The optimal polynomial response surface function is a complete combination of parameters, expressed as

(1) $$f\left(x\right)=a_{0}+a_{1}x_{1}+...+a_{n}x_{n}+a_{n+1}x_{1}^{2}+a_{n+2}x_{1}x_{2}+...+a_{N-1}x_{k}^{m}=\sum_{i=0}^{N-1}a_{i}u_{i}\left(x\right)$$

$u_{i}\left(x\right)$ is the complete polynomial of variable $x=\left(x_{1},x_{2},...x_{n}\right)$; $a_{i}$ is the undetermined coefficient; Eq. (2) is the total number of polynomial terms, defined as

(2) $$N=\frac{\left(n+m\right)!}{n!\cdot m!}$$

Eq. (1) can be transformed into an orthogonal form

(3) $$f\left(k\right)=\sum_{i=0}^{N-1}h_{i}p_{i}\left(k\right)$$

f(k) is the target response value for the $k^{th}$ sampling, k=1,2,...,L; $p_{i}\left(k\right)$ is the orthogonal term under the $k^{th}$ sampling, which can be obtained by orthogonal transformation in Eq. (3); $h_{i}$ is the coefficient of the orthogonal term.

According to the orthogonality of each item in Eq. (3), Eq. (4) can be deduced into

(4) $$\frac{1}{L}\sum_{k=1}^{L}p_{i}\left(k\right)p_{j}\left(k\right)=0$$

By using the Gram-Schmidt orthogonalization process [52], Eq. (5) can be further expressed as

(5) $$p_{i}\left(k\right)=u_{i}\left(k\right)-\sum_{j=0}^{i-1}\alpha_{ij}p_{j}\left(k\right)$$

$u_{i}\left(k\right)$ is the $k^{th}$ sampling polynomial.

And the coefficient $\alpha_{ij}$ Eq. (6) can be expressed as

(6) $$\alpha_{ij}=\frac{\sum_{k=1}^{L}u_{i}\left(k\right)p_{j}\left(k\right)}{\sum_{k=1}^{L}p_{j}^{2}\left(k\right)}$$

The error function *MSE* is defined as

(7) $$MSE=\frac{1}{L}\sum_{k=1}^{L}(f\left(x\right)-\sum_{i=0}^{N-1}{h_{i}p_{i}\left(k\right))}^{2}$$

Take the derivative of $h_{i}$ in Eq. (7), and set it equal to 0, the coefficient of the orthogonal term $h_{i}$ can be expressed as

(8) $$h_{i}=\frac{\sum_{k=1}^{L}f\left(k\right)p_{i}\left(k\right)}{\sum_{k=1}^{L}p_{i}^{2}\left(k\right)}$$

Substituting Eq. (8) into Eq. (5), the error function *MSE* can be reformulated as:

(9) $$MSE=\frac{1}{L}\sum_{k=1}^{L}{\left(f\left(k\right)\right)}^{2}-\sum_{i=1}^{N-1}h_{i}^{2}\left(\frac{1}{L}\sum_{k=1}^{L}p_{i}^{2}\left(k\right)\right)$$

The maximum value of *MSE* is

(10) $$MSE_{\max}=\frac{1}{L}\sum_{k=1}^{L}{\left(f\left(k\right)\right)}^{2}-{\left(\frac{1}{L}\sum_{k=1}^{L}f\left(k\right)\right)}^{2}$$

According to Eq. (9) and Eq. (10), the contribution of orthogonal term $p_{i}\left(k\right)$ to the reduction of *MSE* can be written as

(11) $$C_{MSE}=\frac{1}{L}\sum_{k=1}^{L}h_{i}^{2}p_{i}^{2}\left(k\right)$$

According to Eq. (11), then the error reduction ratio $ERR_{i}$ is defined to quantify the contribution rate of each orthogonal term to the reduction of the error function *MSE*, expressed as

(12) $$ERR_{i}=\frac{100\cdot \sum_{k=1}^{L}h_{i}^{2}p_{i}^{2}\left(k\right)}{\sum_{k=1}^{L}{\left(f\left(k\right)\right)}^{2}-\frac{1}{L}{\left(\sum_{k=1}^{L}f\left(k\right)\right)}^{2}}$$

After the orthogonal transformation, the contribution rate $ERR_{i}$ can be calculated. The largest error reduction rate is selected in the calculation process. The remaining items are orthogonalized according to the above approach, and the contribution rate is evaluated until the maximum $ERR_{i}$ of remaining function items are less than the set threshold. Then the remaining items are discarded. Further the coefficient of reserved term in Eq. (12) can be achieved through the inverse orthogonal transformation, Eq. (13) and Eq. (14) written as

(13) $$a_{i}=\sum_{j=1}^{N-1}h_{j}q_{j}$$

(14) $$q_{j}=-\sum_{r=i}^{j-1}\alpha_{jr}q_{r}$$

When multiple parameters are considered to solve the sensitivity model, the traditional Sobol method based on the response surface function requires high computational cost [53], [54], [55]. Moreover, the reliability problem of the actual engineering equipment structure is more complicated caused by lots of parameters and their coupling effects. To cope with these challenges, an optimal modified polynomial response is constructed through fitting the samples optimized based on Kriging algorithm. Then the sensitivity value can be achieved through the direct integration.

According to Kriging theory [56], [57], [58], [59], the relationship between output response G(x) and input variable *x* can be formulated as

(15) $$G\left(x\right)=f^{T}\left(x\right)\beta +z\left(x\right)$$

In Eq. (15), f(x) is the basis function vector of regression polynomial; *β* is the vector of regression coefficient; z(x) is a Gaussian random process with zero mean. Eq. (16) is defined as

(16) $$\mathrm{cov}\;\left[z\left(x_{i}\right),z\left(x_{j}\right)\right]=\sigma_{z}^{2}\prod_{k=1}^{m}\exp\left[-\theta_{k}{\left(x_{i}^{k}-x_{j}^{k}\right)}^{2}\right]$$

$\sigma_{z}^{2}$ is the variance of Gaussian process; $R\left(x_{i},x_{j},\theta \right)$ is a vector with parameters *θ*, $x_{i}^{k}$ is the kth element of vectors $x_{i}$, $x_{j}^{k}$ is the kth element of vectors $x_{i}$.

Given a set of training samples with capacity *N*, the unbiased estimation and prediction error of G(x) is defined as follows

(17) $$\mu_{G}\left(x\right)=f^{T}\left(x\right)\overset{ˆ}{\beta }+r^{T}\left(x\right)R^{-1}\left(Y-F\overset{ˆ}{\beta }\right)$$

(18) $$\sigma_{G}^{2}\left(x\right)=\sigma^{2}\left[1+u^{T}\left(x\right){\left(F^{T}R^{-1}F\right)}^{-1}u\left(x\right)-r^{T}\left(x\right)R^{-1}r\left(x\right)\right]$$

In Eq. (17) and Eq. (18), $\overset{ˆ}{\beta }$ is the estimated value of *β*; r(x) is the correlation function vector between the training sample point and the prediction point; *Y* is the training sample response

### 1.2 Principle of Sobol global sensitivity analysis

When identifying the model parameters, the *k* dimensional unit volume $\Omega^{k}$ is defined as the spatial domain of the input factor.

(19) $$\Omega^{k}=\{x|0\leq x_{i}\leq 1,i=1,2,...,k\}$$

In Eq. (19), $\Omega^{k}$ is the unit body; $x_{i}$ is the parameter and *k* is the dimension.

According to the Taylor expansion, any function f(x) can be expressed as the sum of sub-items.

(20) $$f\left(x_{1},x_{2},...,x_{k}\right)=f_{0}+\sum_{i=1}^{k}f_{i}\left(x_{i}\right)\;+\sum_{1\leq i<j\leq k}f_{ij}\left(x_{i},x_{j}\right)+\cdot \cdot \cdot +f_{1,2,...,k}\left(x_{1},x_{2},...,x_{k}\right)$$

The total number of decomposition term in Eq. (20) is $2^{k}$. Sobol proposed a decomposition approach using the multiple function integration [60]. The characteristics of Sobol sensitivity algorithm can be summarized as:

➀ $f_{0}$ is a constant term. The integration of each sub-item for any contained factor in Eq. (20) is 0, Eq. (21) can be expressed as

(21) ∫01fi1,i2,...,(xi1,xi2,...,xis)isdxij=0

➁ The sub-items are orthogonal. If $\left(i_{1},i_{2},\cdot \cdot \cdot ,i_{s}\right)\neq \left(j_{1},j_{2},\cdot \cdot \cdot ,j_{l}\right)$ exists, the Eq. (22) must exists.

(22) $$\int_{\Omega^{k}}f_{i_{1},i_{2},\cdot \cdot \cdot i_{s}}\cdot f_{{}^{{}_{j_{1},j_{2},...,j_{s}}}}dx=0$$

The decomposition form of Eq. (3) is unique, and each sub-item can be obtained by multiple integration, Eq. (23) can be written as

(23) $$f_{0}=\int_{\Omega^{k}}f\left(x\right)dx$$

(24) $$f_{i}\left(x_{i}\right)=-f_{0}+\int_{0}^{1}\cdot \cdot \cdot \int_{0}^{1}f\left(x\right)dx_{-i}$$

(25) $$f_{ij}\left(x_{i},x_{j}\right)=-f_{0}-f_{i}\left(x_{i}\right)-f_{j}\left(x_{j}\right)+\int_{0}^{1}\cdot \cdot \cdot \int_{0}^{1}f\left(x\right)dx_{-\left(ij\right)}$$

In Eq. (24) and Eq. (25), there exist 1≤i<j≤k. $x_{-i}$ represents the input parameters other than $x_{i}$; $x_{-\left(ij\right)}$ represents the input parameters other than $x_{i}$ and $x_{j}$.

The total variance of the above model is expressed as Eq. (26).

(26) $$D=\int_{\Omega^{k}}f^{2}\left(x\right)dx-f_{0}^{2}$$

The variance of the sub-terms for each order in Eq. (27) is the partial variance for each order, and its s order partial variance can be expressed as

(27) $$D_{i_{1},i_{2},\cdot \cdot \cdot i_{s}}=\int_{0}^{1}\cdot \cdot \cdot \int_{0}^{1}f_{i_{1},i_{2},\cdot \cdot \cdot ,i_{s}}\left(x_{i_{1}},x_{i_{2}},\cdot \cdot \cdot ,x_{i_{s}}\right)dx_{i_{1}}dx_{i_{2}}\cdot \cdot \cdot dx_{i_{s}}$$

From Eq. (20) and Eq. (27), the relationship between the total variance and the partial variance of each order can be expressed as Eq. (28).

(28) $$D=\sum_{i=1}^{k}D_{i}+\sum_{1\leq i<j\leq j}D_{ij}+\cdot \cdot \cdot +D_{1,2,\cdot \cdot \cdot ,k}$$

The sensitivity coefficient is defined as the ratio of the deviation variance of each order $D_{i_{1},i_{2},\cdot \cdot \cdot ,i_{s}}$ over the total variance *D*, expressed as Eq. (29).

(29) $$S_{i_{1},i_{2},\cdot \cdot \cdot ,i_{s}}=\frac{D_{i_{1},i_{2},\cdot \cdot \cdot ,i_{s}}}{D}$$

$S_{i}$ is the first-order sensitivity coefficient of factor $x_{i}$, which represents the influence of independent condition parameters on the structural reliability; $S_{ij}\left(i\neq j\right)$ is the second-order sensitivity coefficient, which represents the cross-influence between two condition parameters; $S_{1,2,\cdot \cdot \cdot ,k}$ is the *k* order sensitivity, which represents the crossover influences between multi condition parameters.

Response surface function - Kriging model and coefficients of the target response function with respect to the design parameters can be determined by the above model. And the sensitivity of each parameter can be directly calculated by the integration.

**Figure 1:**
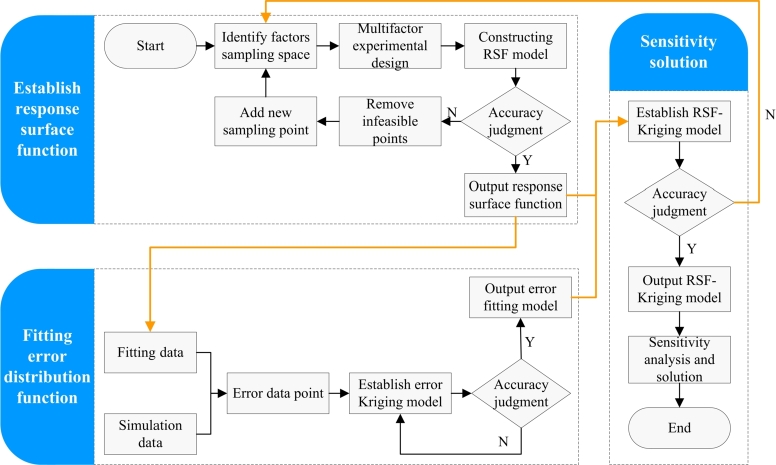
Flow chart for solving RSF-Kriging model.

The flow chart for solving response surface function - Kriging model is shown in Fig. 1, which include mainly three sessions, the construction of response surface function, the error distribution function fitting and the sensitivity analysis.

## 2 Structural reliability analysis of the port crane

### 2.1 Structural reliability analysis based on modified sensitivity model

**Figure 2:**
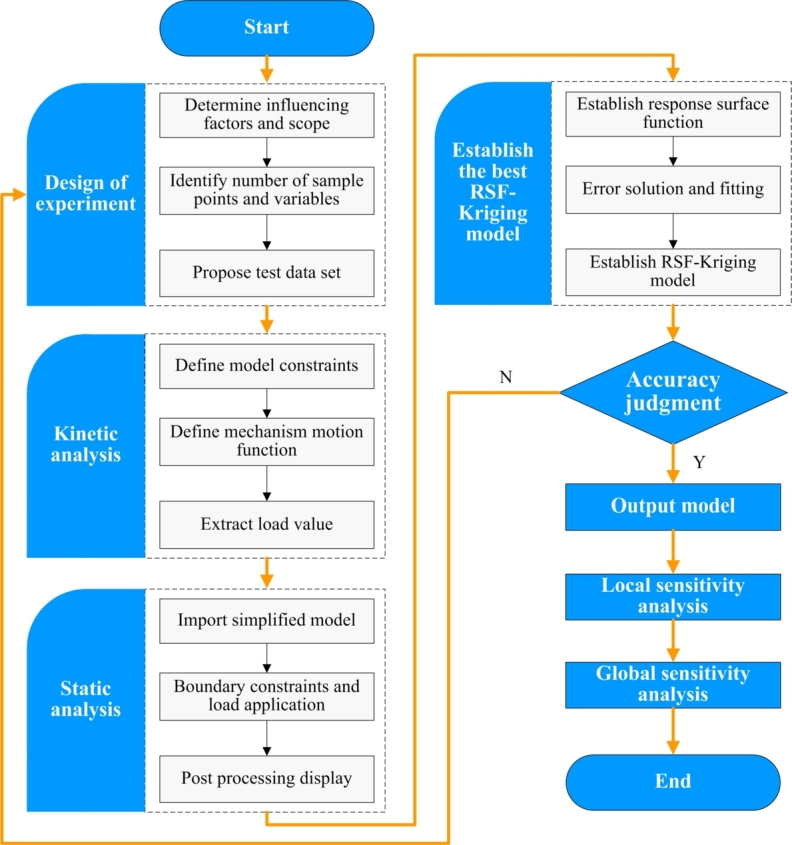
Flow chart of sensitivity analysis of port crane.

The sensitivity analysis using Kriging modified response surface function sensitivity model can be implemented through the flow chart shown in Fig. 2.

The structural influencing parameters, the conventional value range of influencing parameters, the number of sample points and the variable space are determined, based on the working condition analysis. And the experimental scheme of coupled influencing parameters is obtained. Then the load value of each experimental group is solved through the multi-body dynamics simulation, and which further be imported into the structural statics analysis step to complete the static analysis. At the same time, the rationality of the experimental design is evaluated through the range verification. When the assessment is effective, the relevant analysis data is imported into the revised sensitivity model based on Kriging modified response surface function, and the sensitivity of each influencing factor can be analyzed. Then the influencing parameters that affect structural reliability and the cross influence degree can be quantitatively identified.

### 2.2 Structural numerical analysis

#### 2.2.1 Multibody dynamics analysis

**Figure 3:**
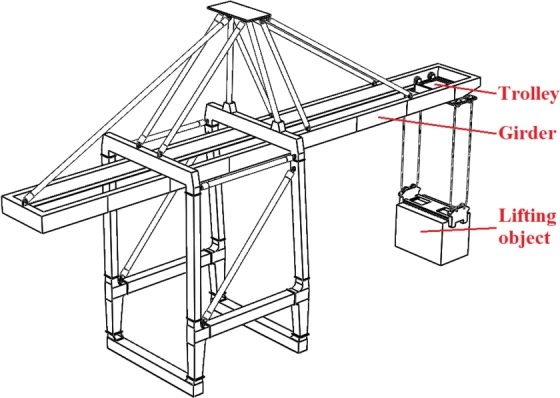
Structural diagram of port crane.

A typical port crane structure is selected to carry out the case study, in order to verify the feasibility and analytical accuracy of the proposed approach. Taking into account of the operating conditions for girder structure, the controllable influencing parameters mainly include [61]: trolley position F_1_, forward speed F_2_, lifting speed F_3_, lifting load F_4_, and sea wind pressure F_5_, as shown in Fig. 3. The influencing parameters above are taken as the experimental parameters, and the maximum equivalent stress of the girder structure is selected as the experimental index.

According to the existing experimental research data, the common value of the controllable working parameters are obtained, shown in Table 1. Using the orthogonal experimental design [62], 49 sets data are determined through the combination of different experimental parameters.

**Table 1:** Design scheme of experimental parameters.

Trolley position F_1_	Forward speed F_2_/(m/s)	Lifting speed F_3_/(m/s)	Lifting load F_4_/(t)	Sea wind pressure F_5_/(N/m^2^)
1	1.26	0.36	0	500
2	1.33	0.42	10	600
3	1.50	0.48	20	700
4	1.67	0.54	30	800
5	1.84	0.60	40	900
6	2.00	0.67	44	1000
7	2.17	0.73	48	1100

**Figure 4:**
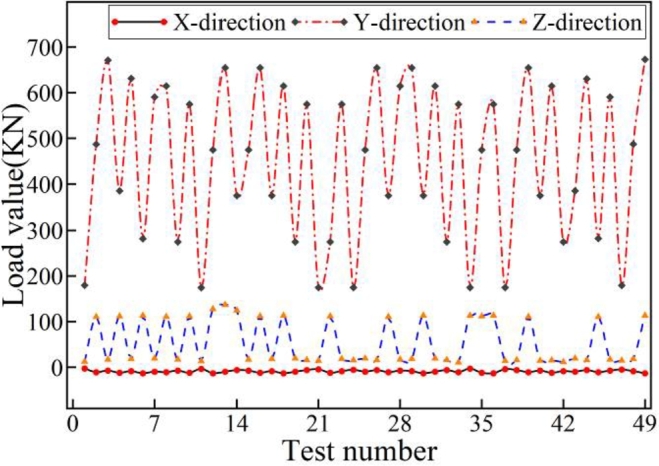
Force components extracted by dynamic simulation.

Under the 49 sets of testing conditions, the multi-body dynamics simulation of the port crane is implemented by using ADAMS software. The reaction force components imposed on the girder structure in the Cartesian coordinates are extracted correspondingly, shown in Fig. 4.

#### 2.2.2 Structural statics analysis

**Figure 5:**
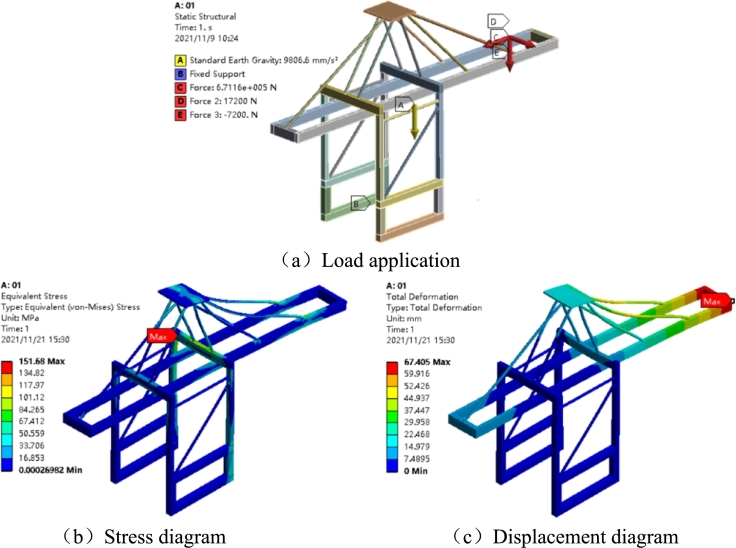
Maximum stress and displacement case.

As shown in Fig. 5 (a), the obtained 49 sets of dynamic reaction forces are imposed on the port crane model, respectively. The material properties of the structure are listed in Table 2.

**Table 2:** The material parameters of port crane.

Material	E/GPa	*σ*_y_/GPa	*σ*_b_/GPa	*ν*
Q235A	206	235	375	0.3

Among the results, the maximum stress and displacement are shown in Fig. 5(b) and Fig. 5(c).

**Figure 6:**
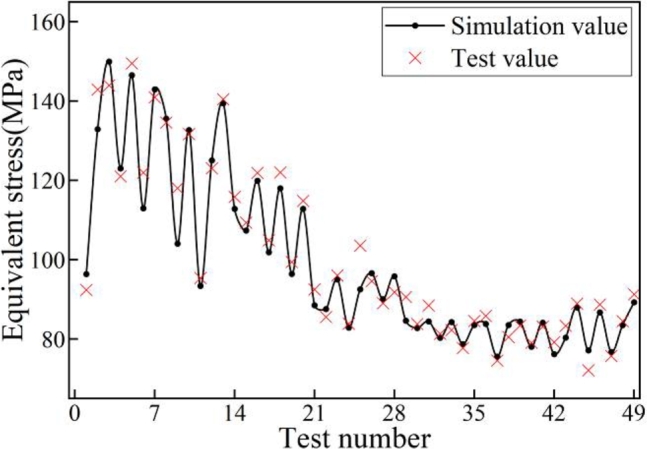
Static simulation and test results.

The simulation and experimental results of 49 sets are shown in Fig. 6. The average numerical prediction is 93.47%.

#### 2.2.3 Experimental range verification

In order to further judge the effectiveness of multi factor test design under different working conditions, range analysis model is used to verify it. When using $L_{n}\left(S^{r}\right)$ to arrange the design scheme, the sum of the solution results of factor *S* in column *j* is $K_{sj}$, $\bar{K_{sj}}$ is the average value of $K_{sj}$, and $R_{j}$ is the range of column *j* factor, that is, the difference between the maximum and minimum value of the index value under each level of column *j* factor.

(30) $$R_{j}=\max\left\{{\bar{K}}_{1j},{\bar{K}}_{2j},...,{\bar{K}}_{sj}\right\}-\min\left\{{\bar{K}}_{1j},{\bar{K}}_{2j},...,{\bar{K}}_{sj}\right\}$$

The larger $R_{j}$ is, the larger impact indicates the parameter. Through the range analysis of the experimental and numerical data shown in Fig. 6, the parameters range are shown in Table 3.

**Table 3:** Range analysis results of influencing parameters.

Factor	Trolley position	Forward speed	Lifting speed	Lifting load	Sea wind pressure
	A	B	C	D	E
*K*_1*j*_	**129.22**	96.50	96.94	84.58	95.76
*K*_2*j*_	120.41	99.72	99.20	90.64	100.07
*K*_3*j*_	106.39	**101.77**	101.55	95.55	97.28
*K*_4*j*_	91.48	96.89	96.73	101.18	**101.65**
*K*_5*j*_	82.65	100.50	**101.65**	105.45	98.96
*K*_6*j*_	80.80	100.22	97.74	107.47	101.06
*K*_7*j*_	83.07	98.43	100.20	**109.15**	99.23
*R*_j_	48.42	5.27	4.92	24.57	5.89
Ranking	1	4	5	2	3

Taking the influences of the fatigue damage as the control target, the trolley position A shall take the 1^st^ level; the lifting load D as the 7^th^ level, the sea wind pressure E as the 4^th^ level, the forward speed B as the 3^rd^ level, and the lifting speed C as 5^th^ level, respectively. And the horizontal combination is $A_{1}D_{7}E_{4}B_{3}C_{5}$. With the comparison of the simulation data, it can be observed that the accuracy of the data is 96.34%, which verifies the effectiveness of the multi factor experimental design.

#### 2.2.4 Establishment of optimal agent model

**Figure 7:**
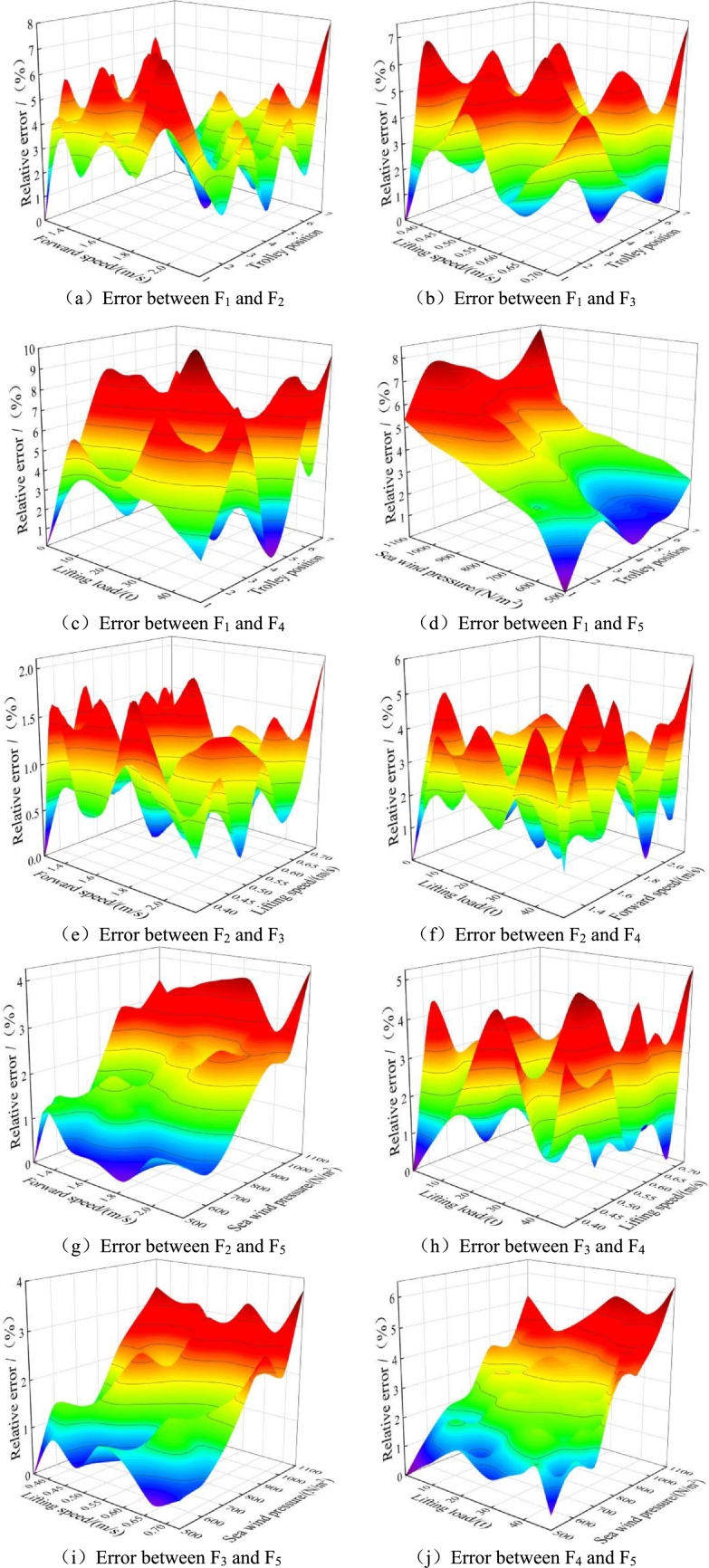
Response surface function error fitting three-dimensional diagram.

According to the results from the multi factor experimental design, the polynomial response surface function between the structural reliability model of port crane and multiple influencing parameters is established, and the error of the proposed preliminary response surface function is fitted based on Kriging optimization algorithm, as shown in Fig. 7. And a modified response surface function - Kriging model is proposed through the integration of the response surface function and its error Kriging fitting model.

Combined with multi factor test analysis, it is obvious that there is a coupling relationship between the influencing parameters of port crane structure. The error values between two influencing factors fitted based on Kriging optimization algorithm can be achieved, as shown in Fig. 7(a-j). The relative error between trolley position and lifting load is the largest, shown in Fig. 7(c), and the relative error between lifting speed and sea wind pressure is the smallest, shown in Fig. 7(i).

**Figure 8:**
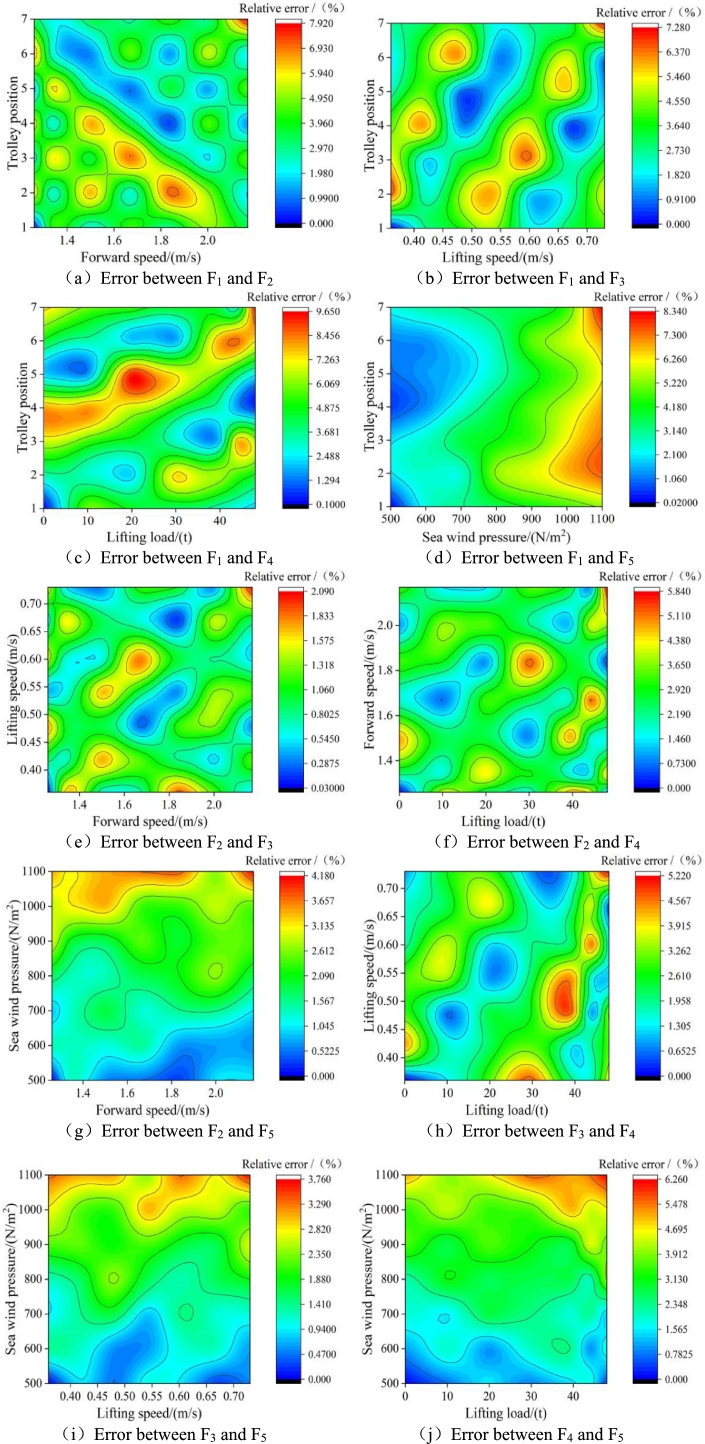
Response surface function error fitting contour diagram.

According to the above solution results, the contour map of the response surface function error between the two of the structural influence parameters of the port crane is obtained, as shown in Fig. 8. The relative error between trolley position and lifting load is the largest. It increases with the increase of lifting load, while it decreases with the increase of trolley position. The relative error between the lifting speed and the sea wind pressure is the smallest, and the degree of change is not so obvious.

## 3 Results and discussion

### 3.1 Local sensitivity analysis

The sensitivity value of different influencing parameters, shown in Table 4, is performed based on the proposed model in Section 2.

**Table 4:** Sensitivity value of corresponding level of each influencing factor.

Different influencing parameters	Corresponding level						
	Level 1	Level 2	Level 3	Level 4	Level 5	Level 6	Level 7
Trolley position	0.4545	0.4178	0.3816	0.3308	0.2429	0.1651	0.1804
Forward speed	0.1131	0.1146	0.115	0.1152	0.1157	0.1167	0.1177
Lifting speed	0.093	0.0936	0.0941	0.0947	0.0952	0.0956	0.0961
Lifting load	0.1204	0.1428	0.1732	0.2408	0.2981	0.3187	0.338
Sea wind pressure	0.1148	0.1174	0.1202	0.123	0.1258	0.1286	0.1314

**Figure 9:**
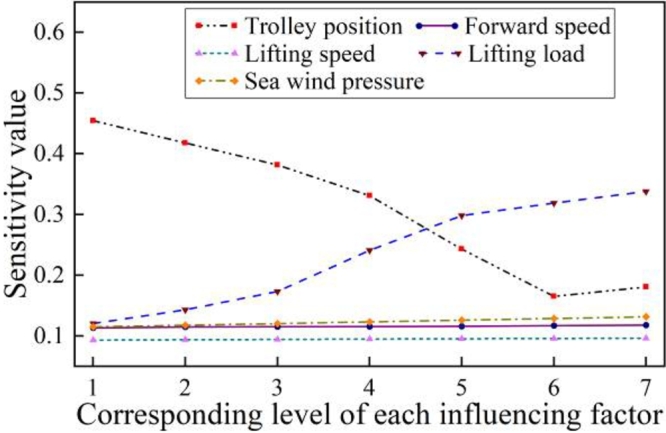
Sensitivity range of single factor.

When single influencing factor is studied, as shown in Fig. 9, with the increase of level change, the sensitivity value is decreasing for trolley position, increasing for lifting load, while almost unchanged for the forward speed, lifting speed and wind pressure.

**Figure 10:**
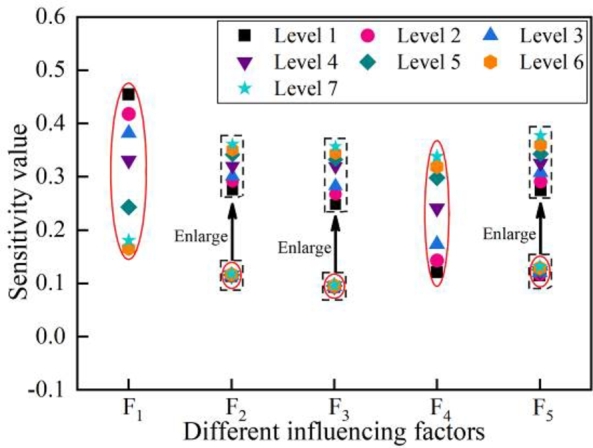
Sensitivity variation range of each factor.

In order to quantify the sensitivity distribution range of the influencing parameters, the results as shown in Fig. 10 can be obtained after the data summarizing. As shown in Fig. 10, the sensitivity value range of the trolley position is the largest, and the sensitivity value range of the lifting speed is the smallest.

### 3.2 Global sensitivity analysis

The local sensitivity analysis is mainly to judge the influence of single factor, but it cannot effectively identify the specific influence degree of coupling relationship among the influencing parameters [63]. So the global sensitivity analysis and the second-order solution result are needed. The global sensitivity coefficients as shown in Table 5 are obtained.

**Table 5:** Global sensitivity value of influencing parameters.

Influence factor	Global sensitivity value
Trolley position	0.5318
Forward speed	0.0501
Lifting speed	0.0494
Lifting load	0.3156
Sea wind pressure	0.0524

**Figure 11:**
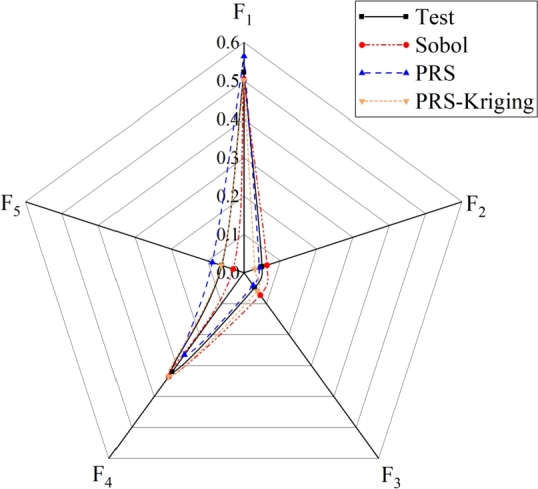
Global sensitivity corresponding to different parameters.

In addition, to further verify the sensitivity and accuracy of the proposed model, a variety of different methods and agent models are used for calculation. The sensitivity value distribution obtained from different models are shown in Fig. 11.

As shown in Fig. 11, the trolley position (F_1_) and lifting load (F_4_) account for a large proportion of the sensitivity, while the forward speed (F_2_), lifting speed (F_3_) and sea wind pressure (F_5_) are not sensitive to the structural reliability.

**Figure 12:**
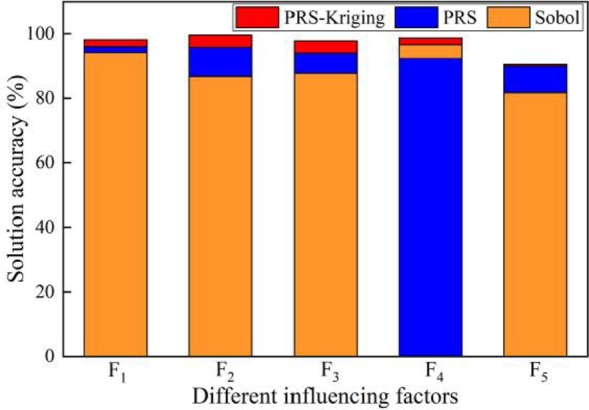
Different parameters correspond to the solution accuracy of different methods.

Then the above solution results can effectively identify the specific influence degree of different parameters. Through the comparative analysis of the sensitivity values obtained by different methods and the test values, the solution accuracy of different methods can be obtained, as shown in Fig. 12. The average accuracy of the proposed agent model is the highest, up to 95.91%.

To study the coupling relations among different parameters, the second-order sensitivity value is computed, as shown in Table 6. $S_{ij}$ denotes the coupling of factor $F_{i}$ and $F_{j}$ (i≠j).

**Table 6:** Second-order sensitivity of two factor coupling.

Coupling factor	Second-order sensitivity value
F_1_&F_2_ → S_12_	0.0055
F_1_&F_3_ → S_13_	0.005
F_1_&F_4_ → S_14_	0.0499
F_1_&F_5_ → S_15_	0.0094
F_2_&F_3_ → S_23_	0.0036
F_2_&F_4_ → S_24_	0.0037
F_2_&F_5_ → S_25_	0.0037
F_3_&F_4_ → S_34_	0.0039
F_3_&F_5_ → S_35_	0.0031
F_4_&F_5_ → S_45_	0.0093

**Figure 13:**
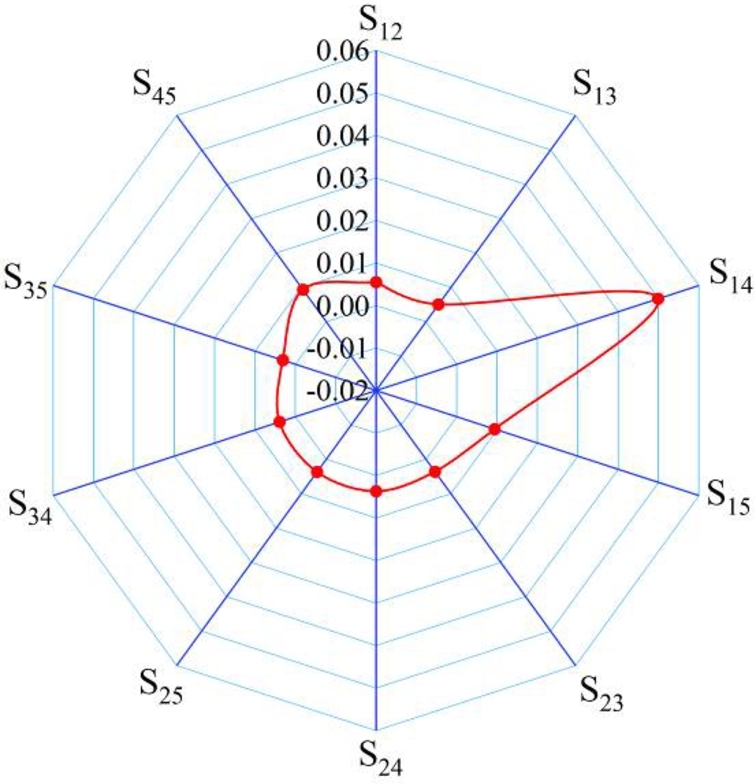
Sensitivity distribution of coupling parameters.

Fig. 13 presents the sensitivity value distribution of the coupling parameters. The coupling degree between the trolley position (F_1_) and the lifting load (F_4_) is the most obvious, and the coupling degree between the lifting speed (F_3_) and the sea wind pressure (F_5_) is the least obvious. The analysis results show that the total order sensitivity index of the analysis object is produced by the interaction between the influencing parameters.

Considering the different degrees of influencing parameters affecting port crane operation under the analysis of global sensitivity, the dangerous operation parameter range is defined as below:

(31) $$\rho_{\min}=-\left|\frac{n-n_{\min}}{n_{\max}-n_{\min}}\right|\cdot \frac{{\left[(1-S_{j})(1-\frac{R_{j}}{\sum_{j=1}^{5}R_{j}})\right]}^{\frac{1}{\lambda }}}{\beta^{\lambda }}\times 100\text{\%}\;\rho_{\max}=\left|\frac{n_{\max}-n}{n_{\max}-n_{\min}}\right|\cdot \frac{{\left[(1-S_{j})(1-\frac{R_{j}}{\sum_{j=1}^{5}R_{j}})\right]}^{\frac{1}{\lambda }}}{\beta^{\lambda }}\times 100\text{\%}$$

$\rho_{\min}$ and $\rho_{\max}$ are the minimum and maximum parameter range of dangerous operation of influencing parameters, respectively. *n* is the most dangerous value of influencing factor parameters, $n_{\min}$ and $n_{m\mathrm{ax}}$ are the minimum and maximum value of influencing factor parameters, respectively. $S_{j}$ is the sensitivity value of influencing parameters; *β* is the number of influencing parameters; $R_{j}$ is the maximum change of the test index when the level of the j-th column factor changes; *λ* is the order number.

**Figure 14:**
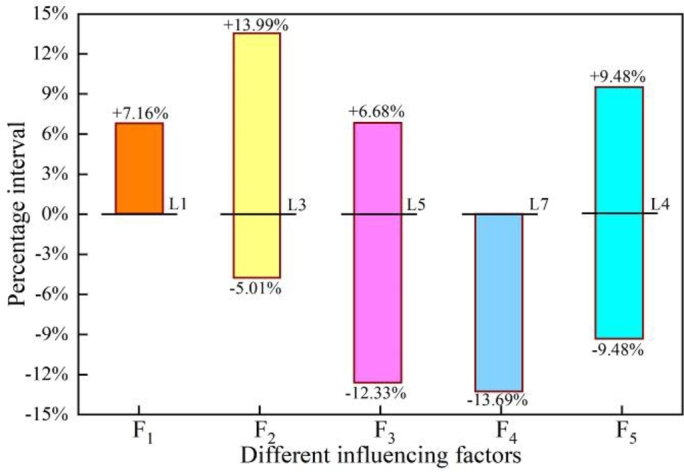
Dangerous working parameter interval of crane under first-order sensitivity correction.

**Figure 15:**
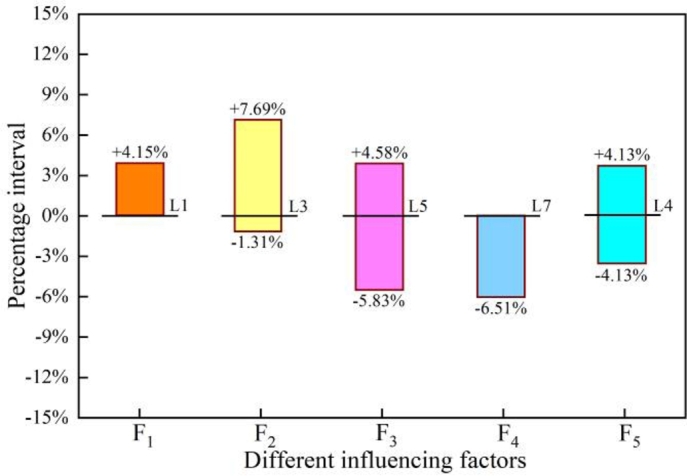
Dangerous working parameter range of crane under second-order sensitivity correction.

Fig. 14 shows the dangerous parameter ranges for different condition parameters. And Fig. 15 illustrates the dangerous range of operation parameters for different condition parameters.

**Figure 16:**
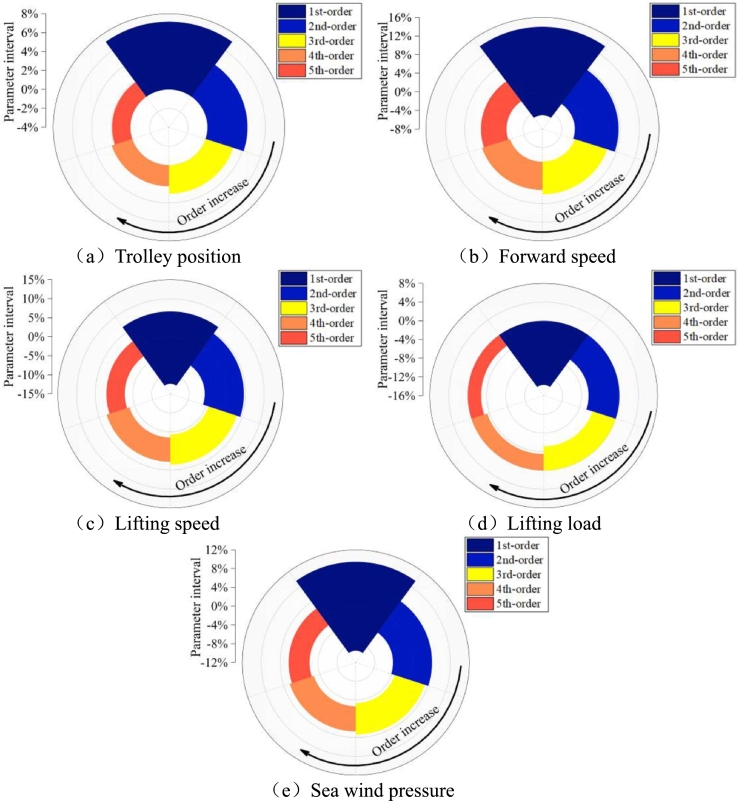
Dangerous working range of different parameters under multi-order sensitivity correction.

According to the above solution method, the distribution of dangerous operating parameters under five order sensitivity revision can be achieved, as shown in Fig. 16. With the increase of the order, the dangerous operating range is gradually reduced. Fig. 16 (a) is trolley position, figure (b) is forward speed, figure (c) is lifting speed, figure (d) is lifting load and figure (e) is sea wind pressure. With the increase of solution order, the range of dangerous operation parameters is gradually refined.

**Figure 17:**
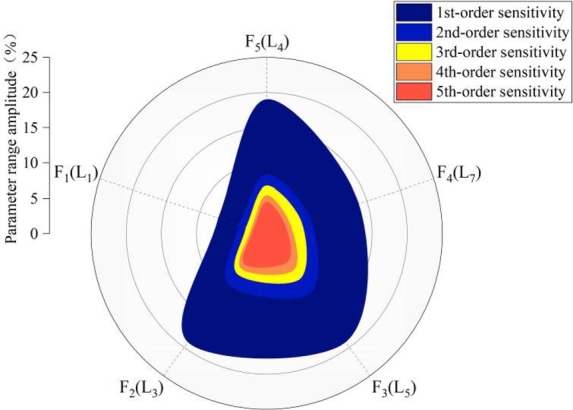
Dangerous operating parameter distribution amplitude under multi-order sensitivity correction.

Further the distribution of dangerous operating parameters amplitude under five order sensitivity revision can be achieved, as shown in Fig. 17. With the increase of the order, the dangerous operating range is gradually reduced. More and more refined operation parameter range is beneficial, from the perspective of protecting the operation safety for equipment structure.

## 4 Conclusion

This paper has developed a revised sensitivity model, which considered the local and global sensitivity analysis of multiple influencing parameters through the integration of the response surface function - Kriging model and the Sobol sensitivity algorithm. Here not only the individual change but also the interaction of factors are investigated.

The proposed approach is illustrated through a practical case crane port with a high prediction accuracy 95.91%. The results reveal that the coupling effect between the trolley position (F_1_) and the lifting load (F_4_) is the highest, and the coupling effect between the lifting speed (F_3_) and the sea wind pressure (F_5_) is the weakest.

The distribution of operating parameters interval are achieved. With the increase of the order, the dangerous value range is gradually reduced. The revised model may provide a good mean for the selection of the dangerous operation parameter range of an industrial equipment.

## Declarations

### Author contribution statement

Zhu Lin: Conceived and designed the experiments; Analyzed and interpreted the data; Contributed materials, analysis tools or data; Wrote the paper.

Qiu Jianchun: Performed the experiments; Analyzed and interpreted the data.

Chen Min, Jia Minping: Analyzed and interpreted the data.

### Funding statement

Min Chen was supported by 10.13039/501100001809National Natural Science Foundation of China [51805447]. Professor Lin Zhu was supported by 10.13039/501100004608Natural Science Foundation of Jiangsu Province [BK20190911]. Professor Lin Zhu was supported by 10.13039/100007540Jiangsu Agricultural Science and Technology Innovation Fund [CX(20)3060]. Professor Lin Zhu was supported by 10.13039/501100010031Postdoctoral Research Foundation of China [2019M661947]. Professor Lin Zhu was supported by the Natural Science Foundation of the Jiangsu Higher Education Institution of China [22KJB460010].

### Data availability statement

Data included in article/supp. material/referenced in article.

### Declaration of interests statement

The authors declare no conflict of interest.

### Additional information

No additional information is available for this paper.
